# Forming national identity with televised cultural rituals: a critical discourse analysis of China’s *Ancient Rhyme and New Voice—Qingming* program

**DOI:** 10.3389/fpsyg.2024.1471431

**Published:** 2024-11-06

**Authors:** Duruo Li, Marwan H. Sallam, Zhiwu He

**Affiliations:** ^1^School of Journalism and Information Communication, Huazhong University of Science and Technology, Wuhan, China; ^2^School of Journalism and Communication, Nanyang Normal University, Nanyang, China; ^3^Faculty of Education, Ibb University, Ibb, Yemen

**Keywords:** critical discourse analysis, cultural rituals, national identity, televised culture, Qingming festival, China

## Abstract

Studies on media and national identity emphasize the need for a comprehensive and culturally sensitive approach to understanding the complex relationship between media and identity. This study used critical discourse analysis to examine the depiction of cultural rituals in the *Ancient Rhyme and New Voice—Qingming* program and its role in forming and reinforcing national identity in China. The program, broadcast on China Central Television, showcases three traditional Chinese cultural rituals: ancestor worship, agricultural rites, and spring outings. The analysis showed that the program aroused shared emotions, beliefs, and values among Chinese audiences, enhancing cohesion and cultural identification, and advancing the construction of Chinese national identity. The study highlights the importance of such cultural programs in promoting cultural identification and underscores the significant role of televised cultural rituals in shaping and reinforcing national identity in an increasingly globalized world.

## 1 Introduction

The pivotal role of broadcast media in molding and reinforcing national identity cannot be overstated. In the age of globalization, the intersection of global media and cultural identity has become increasingly complex, particularly within the diverse ethnic tapestry of societies such as China. Tarling and Gomez (2008) and Jijiao (2016) previously illuminated the nuanced interplay between state policies, urban migration, and cultural identities within China’s multi-ethnic landscape, underscoring the intricate process of fostering unity amidst diversity. With television as a medium in delineating national identity, this study sought to extend this discourse by exploring the complex dynamics in a televised portrayal of cultural heritage (Awagu, 2020; Hartley, 2004; Scriven and Lecomte, 1999). Despite the dual narrative of unifying national identity and fostering diversified cultural citizenship, television’s role in articulating and disseminating national identity narratives remains paramount, particularly regarding cultural heritage programs.

This study conducted a multi-dimensional analysis of the symbolic and communicative practices within *Ancient Rhyme and New Voice—Qingming*’ (*Gǔyùn xīn shēng·qīngmíng*), a program that offers a contemporary window into the ritualistic fabric of Chinese national identity. It premiered on April 5, 2022, on CCTV1 (primary channel of China Central Television [CCTV]) and has been subsequently rebroadcast. The program is a collaboration between the China Media Group and National Cultural Heritage Administration. It is part of the *Ancient Rhyme and New Voice* series, which showcases traditional Chinese festivals, such as the Spring Festival, Dragon Boat Festival, and Mid-Autumn Festival, employing a consistent style and methodology across all episodes. Ancient Rhyme and New Voice—Qingming showcases the Qingming Festival, which is a period dedicated to ancestor worship, agricultural rites, and spring outings. Each program in the series explores the cultural essence, customs, and rituals associated with the respective festival, making the program a representative sample of the series.

Central to our analysis is Carey’s (2008) conceptual dichotomy between the transmission and ritual views of communication. The Ancient Rhyme and New Voice—Qingming program (hereafter program) exemplifies the latter, transforming the act of communication into a shared cultural ceremony that reinforces communal bonds by representing and celebrating shared cultural rituals. The program’s in-depth exploration of Qingming’s cultural essence through performances, interviews, and music underscores the festival’s role in shaping cultural and national identity.

Ritual communication theory further enriches our understanding of media’s role in shaping collective memory and national identity. An et al. (2022) and Sola-Morales (2016) underscored the importance of ritual and drama in the media’s construction of identification, highlighting the significance of ritualization in contemporary secular society. This theoretical framework aids in deciphering the complex relationship between cultural heritage, media representation, and national identity, as discussed by Chambers (2005), Weiss (2007), Waisbord (1998), and Lloyd (2014), highlighting the discursive construction of heritage and identity through media and political recognition. By portraying Qingming as a media ritual, the program fosters a connection between viewers and China’s cultural heritage, encouraging a sense of community and national pride. This perspective is instrumental in comprehending the program’s contribution to cultivating a shared cultural identity and national unity within the context of China’s extensive ethnic diversity.

The integration of traditional Chinese rituals within the program’s modern broadcasting framework reflects a nuanced understanding of cultural heritage as both a mirror and architect of national identity. Scholars such as Liu et al. (2023) and Parker and Song (2009) offer insights into the role of regional media and diaspora communication strategies in shaping and reflecting the multifaceted nature of cultural identities. They frame the analysis of the program within the broader discourse on media’s role in national identity formation in multi-ethnic settings.

Current research into the impact of media on the formation of cultural identity has identified several gaps. Odağ and Hanke (2019) highlighted the necessity for an integrated approach that considers both psychological and communication theories. Shaigerova et al. (2022) underlined the significance of the cultural context in comprehending the role of social media in identity development, while McKenzie (2022) stressed the overlooked importance of culture in the use of digital media among specific ethnic groups. Collectively, these studies indicate the need for a more comprehensive and culturally sensitive approach to unravel the intricate relationship between media and the formation of national identity.

This study aimes to illuminate the intricate relationship between televised cultural heritage and the construction of a cohesive national identity. Through an in-depth analysis of the content, production, and reception of the *Ancient Rhyme and New Voice—Qingming* program, we examine how the televised dramatization of cultural rituals contributes to the formation and reinforcement of national identity in China’s multi-ethnic context. By focusing on the program’s portrayal of ancestor worship, agricultural rites, and spring outings, we explore how these cultural elements resonate with and unite a diverse viewership, thereby strengthening a collective sense of national identity.

## 2 Critical discourse analysis framework

We employed Fairclough’s (1992) critical discourse analysis to examine the program. Fairclough (1992) contended a dialectical relationship between discourse and social structure, mainly that discourse is socially constitutive from the identity, relational, and ideational functions as discourse constructs social identities, social relationships, and systems of knowledge and belief. Discourse analysis is a tool for understanding the processes of construction in discourse (Phillips and Hardy, 2002). As a predominant branch of discourse analysis, critical discourse analysis owes its theoretical heritage to various disciplines. Fairclough et al. (2011) emphasized that critical discourse analysis examines the discursive nature of power relations. They highlighted how discourse constructs cultural ideology throughout history while also emphasizing the interconnectedness between society and text. This methodological choice, influenced by the multimodal analysis advocated by Bednarek (2015), Chepinchikj and Thompson (2016), and Mirhosseini’s (2006) insights into the linkage between text and social contexts, allows us to dissect the complex interplay of cultural heritage and national identity in televised media. Thus, the central research question guiding this analysis is: How does the televised dramatization of cultural rituals in the Ancient Rhyme and New Voice—Qingming program contribute to the formation and reinforcement of national identity in China’s multi-ethnic context?

While our primary focus is on linguistic elements, we also consider visual and musical components due to their importance in the overall meaning-making process (Kress and Van Leeuwen, 2001; Machin, 2010). Fairclough’s (1992) three-dimensional framework encompasses text, discourse practice, and socio-cultural analyses (Fairclough, 1992, 2010). This comprehensive framework, which conceptualizes the program as a microcosm of the broader series, facilitates a deep exploration of the program, particularly focusing on three quintessential cultural rituals: ancestor worship, agricultural rites, and spring outings. By considering the Qingming episode as representative of the series’ overall style, methodology, and themes, this framework allows for a thorough examination of how televised cultural rituals contribute to the formation and reinforcement of national identity in China.

Through text analysis, we scrutinize the linguistic features, visual imagery, and musical elements of the program to uncover how they articulate traditional cultural heritage and its aesthetic nuances (Mayr and Machin, 2023), and is pivotal in understanding how the program’s narrative, visual, and auditory strategies communicate the rich tapestry of cultural practices and values.

Through discourse practice, we seek to unravel the creative processes behind the symbolic representation of culture, examining how they mediate the translation of cultural significance to the audience. We delve into the ways the program’s creators orchestrate and convey cultural symbols, bridging the intangible cultural values with the tangible medium of television presentation (Chouliaraki and Fairclough, 1999).

Finally, our socio-cultural analysis examines the construction of cultural rituals within the media discourse, probing the program’s efficacy in fostering emotional resonance and identity recognition among viewers (Wodak and Meyer, 2015). This dimension is critical in assessing how the program reinforces and promotes national identity among Chinese audiences. Through socio-cultural analysis, we explore the capacity of televised cultural rituals to unite and resonate with a diverse viewership, thus strengthening a collective sense of national identity.

## 3 Methods

### 3.1 Data collection and analysis

We used a complete video recording of the program, which is available on the channel’s platform.^1^ The 1 h and 27 min program was transcribed verbatim, which enabled us to examine it repeatedly, facilitating a deeper and more nuanced analysis (Flick, 2022).

The data analysis process involved multiple close readings of the transcript, followed by video viewing and detailed notetaking. We conducted a thematic analysis, identifying key themes and patterns related to the representation of cultural rituals and the construction of national identity. These themes were then linked and organized into three main categories: ancestor worship, agricultural rites, and spring outings. We also conducted a multimodal analysis, which involved examining the visual and musical elements of the segments to gain a comprehensive understanding of their role in the overall meaning-making process (Rose, 2022). This iterative approach allowed us to systematically examine how the program constructs traditional cultural rituals and how these cultural rituals evoke emotional resonance among the audience, thus promoting cultural identity. Relevant quotes from the program were selected to support the analysis and discussion of the findings. A total of 27 segments encompassing an opening film, host introduction, 14 dialogues, and 13 performances were investigated. Each segment was numbered in order [e.g., Dialogue 01 or Performance 01. To systematically analyze these segments, we developed an analytical matrix (see Supplementary material)]. This matrix allowed us to record, count, and compare various aspects of each segment, including program form, interaction mode, innovation points, cultural symbols, cultural relics, classics, poetry, quotations, cultural connotations, and deep meanings.

The “program form” refers to the performance form, such as song or dance; “interaction mode” describes the various forms of interaction within a segment, such as between the host, guests, and audience; and “innovation points” refer to the innovative ways in which a segment is presented, such as using technology to transform the performers or adopt unique performance styles. Corresponding to the text analysis, the “program form,” “interaction mode,” and “innovation points” were used to scrutinize the language, sound, visuals, artistic form, and aesthetic style of the program.

Corresponding to discourse practice, cultural symbols, cultural relics, classics, poems, and quotations from ancient texts were first recorded for statistical purposes. Next, we researched and studied these elements in detail. Finally, we analyzed the creative process behind the presentation of these cultural elements and studied how the program arranges and conveys cultural symbols.

The “cultural connotations” and “deep meanings” sections documented the core cultural values behind each segment. These core cultural values were derived from the direct expressions of scholars and actors in the program and the researchers’ reflections. Corresponding to the socio-cultural analysis, first, we analyzed the cultural connotations and meanings of each segment. Then, using comparative analysis, we categorized these cultural meanings into the three rituals in question: ancestor worship, agricultural rites, and spring outings. Through this large number of cultural meanings, we systematically examined how the program constructs traditional cultural rituals and how these cultural rituals evoke emotional resonance among the audience, thus promoting cultural identity.

This iterative approach allowed us to identify key themes and patterns related to the representation of cultural rituals and the construction of national identity. These themes were then linked and organized into three main categories: ancestor worship, agricultural rites, and spring outings. Relevant quotes from the program were selected to support the analysis and discussion of the findings (Saldaña, 2021).

This approach was particularly important to explore segments within the program that highlighted cultural relic displays, interviews, and performances. Such segments are vital for understanding the enactment of ancestor worship, agricultural rites, and spring outings, which are central study themes. Therefore, the methodology of utilizing the available video on the channel’s platform, followed by a verbatim transcription and multimodal analysis, provided a comprehensive dataset that was instrumental for our in-depth analysis.

### 3.2 Validation

To ensure analysis credibility, initial findings were reviewed by two researchers with expertise in Chinese traditional culture and critical discourse analysis. Discrepancies were resolved through collaborative discussion to achieve a consensus (Creswell and Miller, 2000). The analysis was supported by detailed descriptions and examples from the program, facilitating readers’ naturalistic generalizations. Theoretical frameworks related to identity, cultural rituals, and media discourse underpin the analysis, enhancing their generalizability and relevance (Tracy, 2010).

## 4 Results and discussion

Our analysis identified three primary dimensions of cultural rituals depicted in the *Ancient Rhyme and New Voice—Qingming* program: worship rituals, spring plowing ritual, and outing ceremony. These dimensions correspond to the three main categories explored in the study: ancestor worship, agricultural rites, and spring outings. Worship rituals emphasize the ‘meticulousness in funeral matters’ and ‘remembering ancestors’, fostering a sense of family and national sentiment. The spring plowing ritual highlights the importance of ‘aligning with nature’ and ‘valuing time’, reinforcing traditional wisdom and work ethic. The outing ceremony showcases the ‘emotions set in landscapes’ and the ‘fusion of joy and sorrow’, emphasizing the Chinese view of nature and life. Together, these dimensions illustrate how the program constructs and reinforces Chinese national identity through televised cultural rituals, evoking shared emotions and cultural identification among diverse Chinese audiences.

### 4.1 Worship rituals: meticulousness in funeral matters, remembering ancestors, and the sentiment of family and country

The Qingming Festival is the most solemn and grand worship festival in China. The “Analect” (*Lúnyǔ*) states, “Meticulousness in funeral matters and remembering ancestors, the virtue of the people returns to its richness”(*Shènzhōngzhuīyuǎn, mín dé guī hòu yǐ*) (Yang, 2009), which expresses the ancient Chinese reverence for departed relatives and ancestors, embodying the core spirit of the Qingming Festival. Ancient Rhyme and New Voice – Qingming presents a panoramic view of the Chinese people’s spiritual heritage of paying tribute to their ancestors and remembering the martyrs, demonstrating the family concept of respecting older adults and the national sentiments intertwined with the nation’s fate.

#### 4.1.1 Respect for ancestors: upholding family teachings by strengthening family relationships

The most important cultural rituals of the Qingming Festival are the various ceremonies to worship ancestors and mourn the deceased. “Meticulousness in funeral matters” (*Shèn zhōng*) and “remembering ancestors” (*Zhuī yuǎn*) signify the filial duties of descendants towards their parents and ancestors in traditional Chinese culture, reminding descendants to be grateful and emulate the virtuous deeds of their forebears. Through a series of worship rituals, the Qingming Festival establishes the concept of respecting older adults and strengthening family relationships while also educating and reminding descendants to remember their origins, practice self-discipline, and act cautiously.

The program traces the formation of the Qingming Festival’s tomb-sweeping customs and explains the cultural significance of respecting ancestors. Professor Kang Zhen from the School of Literature, Beijing Normal University, described the evolution of the Qingming Festival, which merged the two ancient festivals of “*Cold Food”* (*Hánshí*) and “*Shangsi”* (*Shàngsì*) preserving the tradition of the Cold Food Festival’s ancestor worship. Wang Zhan, a deputy researcher at the National Museum of China, showed a Tang Dynasty artifact, a Changsha kiln green glaze brown colored “Cold Food Day without Fire” (*Hánshí yuán wú huǒ*) porcelain pot. The inscription “Birds chirp on new willows, people worship in front of ancient tombs” (*Niǎo tí xīn shàng liǔ, rén bài gǔ fén qián*) reflects the tomb-sweeping tradition of the Cold Food Festival. Beginning in the Tang Dynasty, Qingming tomb-sweeping was incorporated into the national rites, making it an important folk ritual to maintain family and clan harmony and inform people’s morality. The concept of using the strong connection and maintenance of families and clans as the basis for governing the country and society continues to have a significant influence to this day.

Remembering and mourning the deceased is a common sentiment among Chinese people during the Qingming Festival. While observing the falling pear blossoms and empty swings, Nalan Xingde (*Nà lán xìng dé*), a Qing Dynasty poet, wrote the poem *Swing: The Fragile East Wind Breaks the Floating Silk* to express his endless longing for his deceased wife and his sense of loss and desolation. The original program *If Life Were Only as It Seems at First* was inspired by Nalan’s elegiac poem. It used a situational performance and double swing dance to perfectly express the sorrow of being reminded of loved ones by objects and the melancholy of fleeting good times. The stage was visually beautiful, the performance touching, and the artistic conception profound and evocative.

Various Qingming Festival rituals for commemorating ancestors and remembering the deceased have been unceasingly passed down in China, becoming deeply ingrained in the culture and perpetuating the concept of family relationships. These rituals contribute to the stability and harmony of the social order and its widespread reinforcement (Liu, 2010). It not only reflects the habit and deep affection of every ordinary Chinese person but also has an important spiritual force that the people of various ethnic groups in China have consolidated over thousands of years.

#### 4.1.2 Remembering the martyrs: national sentiments unifying people’s hearts

Traditional Chinese culture embodies the moral standards of “filial piety, fraternity, loyalty, and honesty” *(Xiàotì zhōngxìn, lǐyìliánchǐ)* These standards are an extension of one’s respect for elders and care for family members to personal norms of benevolence, righteousness, and sincerity, ultimately evolving into the highest standard of loyalty to the nation. This progression from family to country reflects the Chinese concept of “the family and the country are one” *(Jiā guó yītǐ)*.” The humanistic ideal of “cultivating oneself, managing the family, governing the country, and bringing peace to the world” *(Xiūshēn qí jiā zhìguó píng tiānxià)* further reflects the Chinese spiritual characteristic of actively integrating family emotions with patriotic feelings (Li, 2016). In the Qingming Festival’s worship culture, family worship rituals for paying tribute to ancestors and mourning relatives coexist with collective worship rituals for the nation to remember and commemorate martyrs, thereby highlighting national sentiment.

The program specifically designed an interactive segment to commemorate and remember those who sacrificed their lives for China’s revolution and development. This segment allows viewers to pay tribute to heroes and express condolences. Simultaneously, it displays images of materials that are a part of the collective memory of the Chinese people, such as the image of the *Revolutionary Hero Monument*. Through the Qingming Festival’s ritual of commemorating martyrs, the program bridges history and the present, demonstrating respect and the inheritance of the nation and its history with significant appeal.

“The sentiment of family and country” signifies a high degree of identification with a valued community held by individuals under the influence of traditional Chinese culture (Yang, 2016). The Chinese audience participates in the media ritual of remembering the martyrs during the traditional Qingming Festival, experiencing shared emotions and memories of the country deep within their consciousness. This practice consolidates social forces, enhances cultural belonging, and strengthens the national community’s identification consciousness.

### 4.2 Spring plowing ritual: aligning with nature and valuing time

Chinese traditional culture revolves around “agriculture and human relations” (Liu, 2010), with many festivals created and perpetuated based on farming needs. ‘Qingming’ signifies commencing the spring plowing ritual, which is a critical juncture in the annual cycle of renewal. The program uses the Qingming Festival as a springboard to delve into China’s solar term and farming cultures, highlighting the ancient Chinese wisdom of aligning agricultural activities with the seasons and the spirit of hard work.

#### 4.2.1 Timely farming: following the sequence and passing on wisdom

Tables ‘Qingming’ is one of the 24 solar terms that are a comprehensive set of traditional rituals guiding production and life practices based on natural rhythms, with Qingming marking the “start of farming ceremony.” During this period, rainfall becomes more frequent and the temperature gradually increases. The ancient people used this solar term as a signal for farmers to initiate spring plowing and sowing; hence, the farming adage ‘Around Qingming, plant melons and beans’ became widespread.

Ancient Chinese people observed celestial body movements, recognized changes in seasons, weather, and phenology throughout the year, and divided time into segments: 5 days as one “phenological period,” *(Hòu)* three periods as one “solar term,” *(Jié)* six terms as one “season,”*(Shí)* and four seasons as one “year” *(Suì)*.’ The names of the solar terms, such as “Grain Rain” *(Gǔyǔ)*when rain nurtures grains and “White Dew” *(Báilù)*when dew condenses and turns white, use tangible natural phenomena to denote abstract time. This approach is lively, interesting, and easy to remember and disseminate. The program’s “Twenty-four Solar Terms Song,” performed by a children’s choir, exemplifies the catchy characteristics of China’s solar terms, making it easy to pass on to younger generations. The program also presents the beauty of phenology depicted by the Chinese solar terms through a screen showcasing the 24 solar terms.

In emphasizing the characteristics of the Qingming solar term, Ren Wanping, deputy director of the Palace Museum, presents the earliest existing “Monthly Ordinance” *(Yuè lìng tú)*picture book and guides the audience to appreciate three paintings depicting the three phenological periods of Qingming. The names of these periods, “Tung Tree Begins to Blossom,” *(Tóng shǐ huá)* “Field Mice Transform into Quails,” *(Tiánshǔ huà wéi rú)* and “Rainbows Begin to Appear,” *(Hóng shǐ xiàn)* are poetic. The corresponding illustrations of the blossoming tung tree, newborn chicks, and rainbows in the sky accurately capture the changes in natural phenomena, demonstrating the insight and artistic expression of ancient Chinese people.

The program illustrates how Chinese ancestors transformed complex agricultural calendars into simple life knowledge and vivid artistic expression through knowledge explanation, cultural relic display, and children’s choir performances, among others. It also showcases the tradition of Chinese people eating according to the seasons, using the traditional Qingming dessert “qing tuan” *(Qīng tuán)* as an example. It fosters an appreciation of Du Fu’s “A Spring Night in Happy Rain,” *(Chūn yè xǐyǔ)* illustrating ancient people’s sentiments towards weather changes. It quotes phrases like “Qingming wind” *(Qīngmíng fēng)* and “willow wind” *(Yángliǔ fēng)* to express ancient people’s romantic associations with natural phenology. All of these emphasize the long-standing Chinese agricultural civilization’s focus on the changing natural seasons. This way of farming in harmony with nature has been passed down to the present, forming the unique agricultural civilization and folk wisdom of the Chinese people.

#### 4.2.2 Discarding the old and embracing the new: cherishing time and embracing hard work

“Of the four seasons, spring is the best, and within spring, Qingming is the finest.” As flora revives and fauna is born and begins to grow, and as the call to agricultural labor for the new year resounds, Qingming has always symbolized the philosophy of life that includes shedding the old and welcoming the new, cherishing time and advancing, and welcoming new life and creating the future.

Zhang Zhiqing, deputy director of the National Library of China, quoted *Huainanzi Astronomy Training (Huáinán zi·tiānwén xùn)*: “Fifteen days after the spring equinox, when the Dipper points to the Yi *(Yǐ)* position, the Qingming wind arrives,” meaning all things are renewed. The fresh and clear Qingming wind can nourish new life and encourage people to strive.

The dance program *A Spring Night in Happy Rain* is based on Du Fu’s poem. The poem’s detailed depiction of the spring rain not only expresses an appreciation for the beauty of a rainy spring night and the timely rain that nourishes all things but also values this beautiful time. The program incorporates the gentle and graceful dance of Jiangnan women holding oil-paper umbrellas that bloom a bright color in the hazy spring rain, marrying both intangible and tangible elements of national cultural heritage.

The program also introduces Qingming Festival folk customs, such as folding, inserting, and wearing willows, which originated in the Southern and Northern Dynasties and flourished during the Song Dynasty. The Southern Song Dynasty’s *Old Things in Wulin (Wǔlín jiùshì)* records, “All the houses in the capital are full of inserted willows, even small houses in secluded places are lovely with green.” The saying “planting willows without intention, the willows form a shade” implies that the willow represents the endless hope of spring. Simple rituals like inserting and wearing willows are indicative of placing new hope in new willows, showcasing the unique romanticism of the ancient Chinese.

A year’s plan lies in the spring, and Qingming is the ideal time to start working. People welcome new life in the mild wind and rain of Qingming and start a new year’s struggle with vigorous spring plowing. This spirit of hard work, unremitting progress, cherishing time, and striving forward is deeply engraved in the spirit of the Chinese people.

### 4.3 Outing ceremony: emotions set in landscapes and the fusion of joy and sorrow

Another indispensable folk ceremony of the Qingming Festival is the spring outing. People venture out in droves, immersing themselves in the beautiful spring scenery and engaging in activities such as flying kites, swinging, tug-of-war, and kicking. The program delves deeply into the folk customs and historical culture during the Qingming Festival, appreciating the dual implications of humanistic and natural aspects of the Qingming Festival and showcasing the unique Chinese view of nature and life.

#### 4.3.1 Outing for a spring walk: harmonious and balanced view of nature

After hibernating during winter, people thrived during the Qingming period, when the weather was ideal for outings. Taking family members to appreciate the landscape, young men and women encountering each other while bathing by the water after tomb-sweeping, literati reciting poetry, and elegant gatherings were all rich elements of the Qingming spring outing in ancient times.

The program introduces the Qingming outing tradition from the traditional ceremony of the *Shangsi Festival–Fu Xi (Fú xì)*, where the ancients went to the water to bathe on the third day of lunar March, washing away the bad luck from their bodies and embracing spring with a renewed outlook. The program appreciates and musically interprets Du Fu’s *Beautiful Lady*, Ouyang Xiu’s *Picking Mulberry: Qingming Shangsi West Lake is Charming*, and Qin Guan’s *Walking Incense: Trees Surround the Village*, showcasing the beauty of the pastoral scenery and the grandeur of the Qingming spring outing of the ancients.

During the Tang Dynasty, the Qingming spring outings were more prosperous. The program showcases the earliest existing scroll landscape painting in China, Zhan Ziqian’s green landscape painting, *Spring Outing (Yóuchūntú)*. The picture depicts lakes and mountains, scholars riding horses on mountain paths, ladies boating on the water, and green mountains. Building on this image, the program created the dance *Spring Outing*, which features more than a dozen graceful and lively Tang Dynasty court ladies enjoying the view and dancing in the wild. The female dancers changed their costumes twice, replicating the *Tang Dynasty Painted Dancing Girl* figurine that can be found in the collection of the Xinjiang Uygur Autonomous Region Museum and the *Tang Dynasty Gold-Painted Pottery Woman* figurine that is part of the collection of the Shanghai Museum. The costumes and hairstyles are meticulously replicated, and the movements and expressions are identical.

Ancient literati have a tradition of reciting poetry, drinking tea, and playing the “Qin” *(Qín)* at elegant gatherings during Qingming. The program *Sudi Spring Dawn* captures the natural scenery of blooming flowers in the Qingming season and the cheerful atmosphere of the literati and scholars bonding over music and enjoying spring through a folk music ensemble. The program *Looking at Jiangnan* recreates the refined daily life of ancient literati brewing and tasting tea through poetry recitation and a performance of one of the *Four Elegances* of the Song Dynasty tea ceremony, revealing the perfect integration of nature and humanities in traditional culture through tea culture.

Among traditional Chinese festivals, the Qingming Festival is the one during which people interact most closely with nature. To express their emotions about the natural landscape, they would paint landscapes and compose poems about nature. This love for nature stems from the ancients’ yearning for freedom and their awareness of life’s return, shaping the Chinese ecological concept advocating harmonious coexistence and balanced development between man and nature.

#### 4.3.2 Outdoor games: an optimistic and open-minded view of life

The Qingming Festival often carries a sense of melancholy, encapsulating the heaviness of paying tribute to the deceased, homesickness—as encapsulated in the phrase, “Guests do not miss home during Qingming”—and the sorrow of parting and gifting willows. Concurrently, the Qingming Festival is also associated with many joyful memories such as flying kites and playing football. Chinese traditional culture possesses not only a rich and profound side but also a light and lively one. During the Qingming Festival, we honor our ancestors but also look forward to the future, strive for progress, and enjoy life, embodying the optimistic and open-minded Chinese attitude towards life.

Kang Zhen uses well-known Qingming poems to illustrate this attitude. The first two lines of *Qingming* by Tang Dynasty poet Du Mu, “In the Qingming season, the rain is continuous, and the pedestrians on the road want to break their souls,” reflect the sadness of some people who may feel disheartened after tomb-sweeping. The following lines, “Ask where the wine shop is, the shepherd boy points to the apricot flower village from afar,” which the poet wrote while observing the boundless spring colors, dispels the sadness with the wish to borrow good wine for solace. The open-mindedness is vividly captured in the paper. The program expresses the artistic conception of this poem by blending it with the scene through singing and folk music performances, leaving a lasting impression.

Another example is Su Shi’s *Looking at Jiangnan*, which begins with a sigh because of homesickness, wine drinking, and sadness. By starting a new fire and drinking the new tea of Qingming, however, the mood suddenly shifts from the melancholy of the past to cherishing the present and longing for the future, “Stop thinking about old friends and old countries and try new tea with a new fire. Poetry and wine take advantage of the years.” The program *Looking at Jiangnan* presents the poet’s expansive and sublime mood with poetry chanting and the tea art performance of the *Seven Soup Tea Method (Qī tāng diǎn chá fǎ).*

The Qingming Festival boasts a rich assortment of outdoor folk customs (e.g., kite flying). Zhang Zhiqing cites the record from the ancient book *Emperor’s Annual Record of Victory (Dìjīng suì shí jì shèng)*from the Qing Dynasty, which reveals that during the Qingming Festival, “all the men and women in the city venture to the suburbs” to leave the city, sweep tombs, and carry paper kites and reels. Once the tomb-sweeping concludes, they initiate a competition in front of the tomb. Amid the sacrifice, people are consumed by grief but set aside their somber moods and compete in kite-flying in front of the tomb. When the ritual concludes, they hope that all the illnesses and misfortunes of the past year will vanish with the wind.

Another example is the tug-of-war sport, which originated in the spring and autumn period and evolved from strength training. Emperor Xuanzong of the Tang Dynasty was particularly fond of tug-of-war. He once organized a tug-of-war event for thousands of people during the Qingming Festival. The verse “The long rope binds the sun, and the threaded rope pulls the river flow” encapsulates the grand scene from that time. The Tang Dynasty not only incorporated Qingming tomb-sweeping into the national ceremony but also transformed Qingming tug-of-war into a grand court game. The ancients did not perceive deep mourning and sweeping as fundamentally opposite to the enthusiastic enjoyment of the present. The program highlights the intriguing scenes of these recreational activities with the song *Paper Kite* and the dance *Tug of War,* making it entertaining and captivating.

These rich and diverse Qingming entertainment folk customs are quite engaging and offer enjoyment for all, from emperors and nobles to ordinary people. It fully demonstrates that the Qingming Festival closely links and integrates the past with the present, the living with the deceased, nature with humanities, rituals with daily life, and the court with the folk. The Qingming Festival promotes reverence for history and encourages remembrance of the past even while cherishing the present and loving life, which bears profound implications. It carries the Chinese people’s philosophical thoughts on life and displays enthusiasm and open-mindedness, embodying their aspirations and hopes for each new year. The program vividly recreates the rich outdoor entertainment activities of the Qingming Festival and explicates the deep characteristics of Chinese culture’s “harmony of sorrow and joy” *(Yōu lè yuán róng)* (Pang, 2014). This optimistic and vigorous national character inspires the Chinese people to remember history and muster the courage to continue crafting a brand-new future.

### 4.4 Limitations and directions for future research

This study has some limitations. Although the program serves as a representative sample of the broader series, future research could analyze multiple programs or compare different cultural heritage programs to provide a more comprehensive understanding of the relationship between televised cultural rituals and national identity formation. Importantly, this study relied primarily on qualitative methods and did not examine the audience’s reception of the program. We acknowledge that without direct audience data, we cannot definitively claim that these programs contribute to increasing national identity. Our analysis instead focuses on how the program constructs and presents cultural rituals with the potential to influence national identity formation. Future studies should address this limitation by investigating audience reception among diverse Chinese audiences, which would provide concrete data on the actual impact of these programs on viewers’ sense of national identity. Additionally, researchers could (1) comparatively analyze different cultural heritage programs and explore the role of social media in disseminating and discussing such programs and investigate audience reception among diverse Chinese audiences, and (2) include other forms of media and engage in cross-cultural comparisons of televised cultural rituals and their role in national identity formation. By pursuing these avenues, researchers can further understand the complex interplay between media, cultural rituals, and national identity in China and beyond.

## 5 Conclusion

Employing Fairclough’s (1992) three-dimensional framework, the critical discourse analysis of the program revealed how televised cultural rituals may contribute to the formation and reinforcement of national identity in China. The showcasing of sacrificial offerings, agricultural rituals, and spring outing rituals artistically depicts Chinese attitudes towards ancestors, the nation, festivals, labor, nature, and life, eliciting emotional resonance among the vast Chinese audience.

The program serves as an innovative practice for communicating traditional cultural knowledge, providing aesthetic appreciation, and interpreting as well as sharing Chinese traditional cultural rituals. It is instrumental in arousing shared emotions, beliefs, and values among local Chinese audiences and overseas Chinese viewers. Consequently, it appears to enhance cohesion and cultural identification and advances the construction of Chinese national identity.

Cultural programs themed on traditional festivals provide a form of ceremonial communication practice that promotes cultural identification. By integrating traditional cultural resources, such as relics, classics, poetry, music, and dance, these programs interpret the cultural connotations of traditional festivals in multiple dimensions. Moreover, they effectively establish the internal associations of a cultural community and promote cultural identification.

In the context of globalization and new media environments, where the complex interweaving of various cultural concepts and media phenomena poses a challenge to the sense of community in multi-ethnic countries, traditional festivals and television programs play a vital role in establishing a national cultural identity. Through cultural rituals, these programs provide a cultural gathering point for members of a national community, affirming cultural identities and activating shared memories and emotions. Celebrating and expressing these shared cultural rituals becomes a means of promoting unity and resisting cultural invasion and assimilation.

Furthermore, traditional festivals and television programs employ the highly popular format of television cultural rituals, which introduces younger generations to specific cultural customs, such as traditional music, calligraphy, painting, and ancient poetry. This approach maintains the modern vitality of traditional culture and preserves cultural heritage.

Our study highlights the potential role of televised cultural rituals in shaping and reinforcing national identity in China, though further audience studies are needed to confirm this. By showcasing traditional cultural practices and values, the program fosters cultural identification and unity among Chinese audiences, underscoring the importance of media in the construction and maintenance of national identity in an increasingly globalized world.

## Data Availability

The original contributions presented in the study are included in the article/Supplementary material, further inquiries can be directed to the corresponding author.

## Footnotes

^1^The program’s video is available at the following link: https://tv.cctv.com/2022/06/20/VIDE9slion5kCtg8DiclgFwt220620.shtml
